# Detection of Serum Antibodies Cross-Reacting with *Mycobacterium avium* Subspecies *paratuberculosis* and Beta-Cell Antigen Zinc Transporter 8 Homologous Peptides in Patients with High-Risk Proliferative Diabetic Retinopathy

**DOI:** 10.1371/journal.pone.0107802

**Published:** 2014-09-16

**Authors:** Antonio Pinna, Speranza Masala, Francesco Blasetti, Irene Maiore, Davide Cossu, Daniela Paccagnini, Giuseppe Mameli, Leonardo A. Sechi

**Affiliations:** 1 Department of Surgical, Microsurgical and Medical Sciences, Section of Ophthalmology, University of Sassari, Sassari, Italy; 2 Department of Biomedical Sciences, Section of Experimental and Clinical Microbiology, University of Sassari, Sassari, Italy; 3 Azienda Ospedaliero-Universitaria di Sassari, Sassari, Italy; Massachusetts Eye & Ear Infirmary, Harvard Medical School, United States of America

## Abstract

**Purpose:**

MAP3865c, a *Mycobacterium avium* subspecies *paratuberculosis* (MAP) cell membrane protein, has a relevant sequence homology with zinc transporter 8 (ZnT8), a beta-cell membrane protein involved in Zn++ transportation. Recently, antibodies recognizing MAP3865c epitopes have been shown to cross-react with ZnT8 in type 1 diabetes patients. The purpose of this study was to detect antibodies against MAP3865c peptides in patients with high-risk proliferative diabetic retinopathy and speculate on whether they may somehow be involved in the pathogenesis of this severe retinal disorder.

**Methods:**

Blood samples were obtained from 62 type 1 and 80 type 2 diabetes patients with high-risk proliferative diabetic retinopathy and 81 healthy controls. Antibodies against 6 highly immunogenic MAP3865c peptides were detected by indirect ELISA.

**Results:**

Type 1 diabetes patients had significantly higher rates of positive antibodies than controls. Conversely, no statistically significant differences were found between type 2 diabetes patients and controls. After categorization of type 1 diabetes patients into two groups, one with positive, the other with negative antibodies, we found that they had similar mean visual acuity (∼0.6) and identical rates of vitreous hemorrhage (28.6%). Conversely, Hashimoto's thyroiditis prevalence was 4/13 (30.7%) in the positive antibody group and 1/49 (2%) in the negative antibody group, a statistically significant difference (*P* = 0.016).

**Conclusions:**

This study confirmed that type 1 diabetes patients have significantly higher rates of positive antibodies against MAP/ZnT8 peptides, but failed to find a correlation between the presence of these antibodies and the severity degree of high-risk proliferative diabetic retinopathy. The significantly higher prevalence of Hashimoto's disease among type 1 diabetes patients with positive antibodies might suggest a possible common environmental trigger for these conditions.

## Introduction

Diabetes mellitus is the most common endocrine disorder in industrialized countries. Two main forms are recognized [1]. Type 1 diabetes (T1D) is due to a deficiency in endogenous insulin secretion secondary to destruction of insulin-producing beta cells in the pancreas. Although T1D does have a peak incidence around the time of puberty, approximately 25% of cases present after 35 years of age. Type 2 diabetes (T2D) is characterized by insulin resistance with an insulin secretory defect leading to relative insulin deficiency. This group accounts for 90–95% of patients with diabetes and also has a strong genetic predisposition. T2D patients are usually, but not always, older than age 40 at presentation. Obesity is a frequent finding and, in the United States, is present in 80–90% of these patients.

Diabetic retinopathy is the leading cause of new cases of legal blindness among working-age people in developed countries. Proliferative diabetic retinopathy (PDR), the most vision-threatening form of diabetic retinopathy, has been reported to be present in approximately 50% of T1D patients with 25 years' duration of the disease and in 25% of patients who have had T2D for 25 years or more [2], .

Zinc transporter 8 (ZnT8), a pancreas beta-cell membrane protein involved in Zn++ transportation, may act as a major autoantigen in T1D [4]. Auto-antibodies against ZnT8 have been found in 60–80% of newly-diagnosed cases of T1D [5]. Furthermore, dysregulation in Zn++ homeostasis caused by ZnT8 downregulation has been implicated in the pathogenesis of ischemic retinopathy [6]. *Mycobacterium avium* subspecies *paratuberculosis* (MAP) is transmitted from dairy herds to humans through food contamination. MAP causes an asymptomatic infection that is highly prevalent in patients with T1D, compared to those with T2D and healthy controls [7], [8]. MAP3865c, a MAP cell membrane protein, has been shown to display a relevant sequence homology with ZnT8 [4], [9]. Moreover, antibodies recognizing MAP3865c epitopes have been found to cross-react with ZnT8 in T1D patients [4], [9], [10].

We are unaware of any former study investigating a possible role of auto-antibodies against MAP/ZnT8 epitopes in the pathogenesis of PDR. The purpose of this study was to detect antibodies against 6 highly immunogenic MAP3865c peptides in patients with high-risk PDR, the most severe form of PDR, and in healthy controls and speculate on whether, or not, these antibodies may somehow be involved in the pathogenesis of PDR.

## Methods

### Patients and Controls

The present study used a case-control design, recruiting 62 T1D and 80 T2D patients with high-risk PDR and 81 healthy controls, all accrued between January and December 2013.

The inclusion criteria for the case group were diagnosis of T1D or T2D with high-risk PDR and age ≥18 years. Both newly-diagnosed cases of high-risk PDR and well-established cases, already treated with retinal laser photocoagulation, were included. According to the Early Treatment of Diabetic Retinopathy Study (ETDRS) classification, the diagnosis of high-risk PDR was made by the detection of new vessels on or within one disc diameter of the optic disc equaling or exceeding standard photograph 10A (about 1/4 to 1/3 disc area), with or without vitreous or preretinal hemorrhage; or vitreous and/or preretinal hemorrhage accompanied by new vessels either on the optic disc less than standard photograph 10A or new vessels elsewhere equaling or exceeding 1/4 disc area on ophthalmoscopic examination and fluorescein angiography [11], [12]. Plasma glucose, creatinine, and glycated hemoglobin (HbA_1c_), and medical conditions, including body mass index (BMI), systemic hypertension, hypercholesterolemia, diabetic nephropathy, peripheral neuropathy, and cardio- and cerebrovascular status were recorded. All diabetic patients underwent a full ophthalmic evaluation, including best corrected visual acuity (BCVA), slit-lamp examination, applanation tonometry, fundus biomicroscopy, and fluorescein angiography. Exclusion criteria included any level of non-Sardinian ancestry and evidence of any other retinal vascular disorder.

Apparently healthy subjects, recruited from accompanying relatives or friends of patients or from hospital personnel, were used as controls. Exclusion criteria included clinical/laboratory evidence of diabetes mellitus, age <18 years, any level of non-Sardinian ancestry, and previous history of retinal artery occlusion, retinal vein occlusion, or anterior ischemic optic neuropathy. All controls underwent standard ophthalmic evaluation, including BCVA, slit-lamp examination, applanation tonometry, and fundus examination. Plasma glucose, systolic and diastolic blood pressure, and medical conditions were also recorded.

Subjects were classified as diabetic if they were under treatment for T1D or T2D or if they had a fasting plasma glucose level of ≥126 mg/dL and/or a plasma glucose level of ≥200 mg/dL 2 hours after a 75-g oral glucose load in a glucose tolerance test (as defined by the WHO). Subjects were considered to have hypertension if they were receiving treatment with anti-hypertension drugs or if their blood pressure was >140 mm Hg systolic or >90 mm Hg diastolic (as defined by the WHO/International Society of Hypertension). Hypercholesterolemia was defined by a fasting plasma cholesterol level of ≥200 mg/dL or the intake of lipid-lowering drugs.

Approval from the Ethics Committee/Institutional Review Board of the Department of Surgical, Microsurgical, and Medical Sciences, University of Sassari, Sassari, Italy, was obtained and the study was conducted in full accord with the tenets of the Declaration of Helsinki. Each participant received detailed information and provided written informed consent before inclusion.

Eight percent of the cases and 10% of the controls who were eligible for the study declined to participate. The major reason was “not interested”.

Categorical values were compared by Chi-square test. The differences between cases and controls for quantitative variables were analyzed by Student's *t* test or Mann-Whitney test, when appropriate.

### Blood collection

A blood sample was taken from each participant. Five ml of peripheral blood was collected in Vacutainer Serum tubes, transported at room temperature, left to clot for 15–30 minutes, and finally centrifuged at 2000×*g* for 10 minutes for plasma and serum separation. The supernatant serum was removed, stored at −80°C, and analyzed within 6 months.

### Peptides

Six MAP/ZnT8 peptides were synthesized at >90% purity (LifeTein, South Plainfield, NJ 07080, USA). Four fell within the transmembrane region [MAP3865c_125–133_ (MIAVALAGL), MAP3865c_133–141_ (LAANFVVAL), MAP3865c_246–252_ (LSPGKDM), MAP3865c_256–262_ (HLISTGD)] and two were homologues to the human C-terminal ZnT8 immunogenic region [MAP3865c_261–267_ (GDSARVL) and MAP3865c_281–287_ (HATVQID)].

### Enzyme-Linked Immunosorbent Assay (ELISA)

Antibodies specific for MAP3865c peptides were detected by indirect ELISA, as described previously [9]. Receiver operating characteristic (ROC) curves were used to calculate the cut-off values for positivity. The specificity was set at 93.8% (i.e. Ab + healthy controls <6.2%). The Area Under ROC Curve (AUC) and the percent fraction of antibody + sera were determined. Results were assessed by Fisher exact test and *P* values ≤0.05 were considered to be statistically significant. Statistical analysis was performed with commercial software (Graphpad Prism 6.0). ELISA results were normalized to a strongly positive control serum tested in all experiments, whose reactivity was set at 10.000 arbitrary units (AU)/ml. ELISA precision was determined by calculating both the inter- and intra-assay coefficients of variation (CV).

## Results

The study group consisted of 62 T1D patients (mean age: 48.6±13.5 years; mean diabetes duration: 32.1±9.9 years) and 80 T2D patients (mean age: 66.6±7.4 years; mean diabetes duration: 15.1±8.5 years), all with bilateral high-risk PDR. The systemic characteristics of T1D and T2D patients are reported in Table 1. Both had similar median values of plasma glucose, creatinine, and glycated hemoglobin and similar rates of systemic hypertension, hypercholesterolemia, diabetic nephropathy, peripheral neuropathy, and Hashimoto's thyroiditis. Conversely, mean BMI was significantly higher in T2D patients, thus confirming that obesity is a frequent finding in T2D. Median glycated hemoglobin was 7.7 (61 mmol/mol) in T1D patients and 7.2 (55 mmol/l) in T2D patients, a result indicating poor glycemic control.

**Table 1:** Systemic characteristics of T1D and T2D patients with high-risk proliferative diabetic retinopathy (PDR).

	T1D patients	T2D patients	*P*
	(n = 62)	(n = 80)	T1D vs. T2D
**Age, years, mean ± S.D.**	8.6±13.5	66.6±7.4	<0.0001^a^
**Gender**			
**- men, n. (%)**	31 (50)	51 (63.8)	0.5^b^
**- women, n. (%)**	31 (50)	29 (36.2)	0.4^b^
**Body mass index, mean ± S.D.**	25.4±4	28.5±4.8	0.0001^a^
**Diabetes duration, years, mean ± S.D.**	32.1±9.9	15.1±8.5	<0.0001^a^
**Plasma glucose, mg/dL, median (95% C.I.)**	155 (150–171)	149 (140–160)	0.288^c^
**Plasma creatinine, mg/dL, median (95% C.I.)**	0.85 (0.79–0.91)	0.85 (0.76–0.9)	0.822^c^
**HbA_1c_, %, median (95% C.I.)**	7.7 (7.4–8)	7.2 (6.8–8.2)	0.074^c^
**HbA_1c_, mmol/mol, median (95% C.I.)**	61 (57–64)	55 (51–66)	0.074^c^
**Systemic hypertension, n. (%)**	32 (51.6)	58 (72.5)	0.25^b^
**Hypercholesterolemia, n. (%)**	26 (41.9)	40 (50)	0.63^b^
**Diabetic nephropathy, n. (%)**	1 (1.6)	5 (6.3)	0.36^b^
**Peripheral neuropathy, n. (%)**	3 (4.8)	0 (0)	0.18^b^
**Hashimoto's thyroiditis, n. (%)**	5 (8)	1 (1.3)	0.14^b^

a Student's *t* test

b Chi-square test

c Mann-Whitney test

The control group consisted of 81 healthy subjects (39 men, 42 women; mean age: 48±12 years).

ELISA intra-assay CV ranged from 5.3% to 6.8%, whereas inter-assay CV ranged from 7.5% to 8.1%. These low CV values confirm that the results were consistent throughout the study.

**Figure 1:**
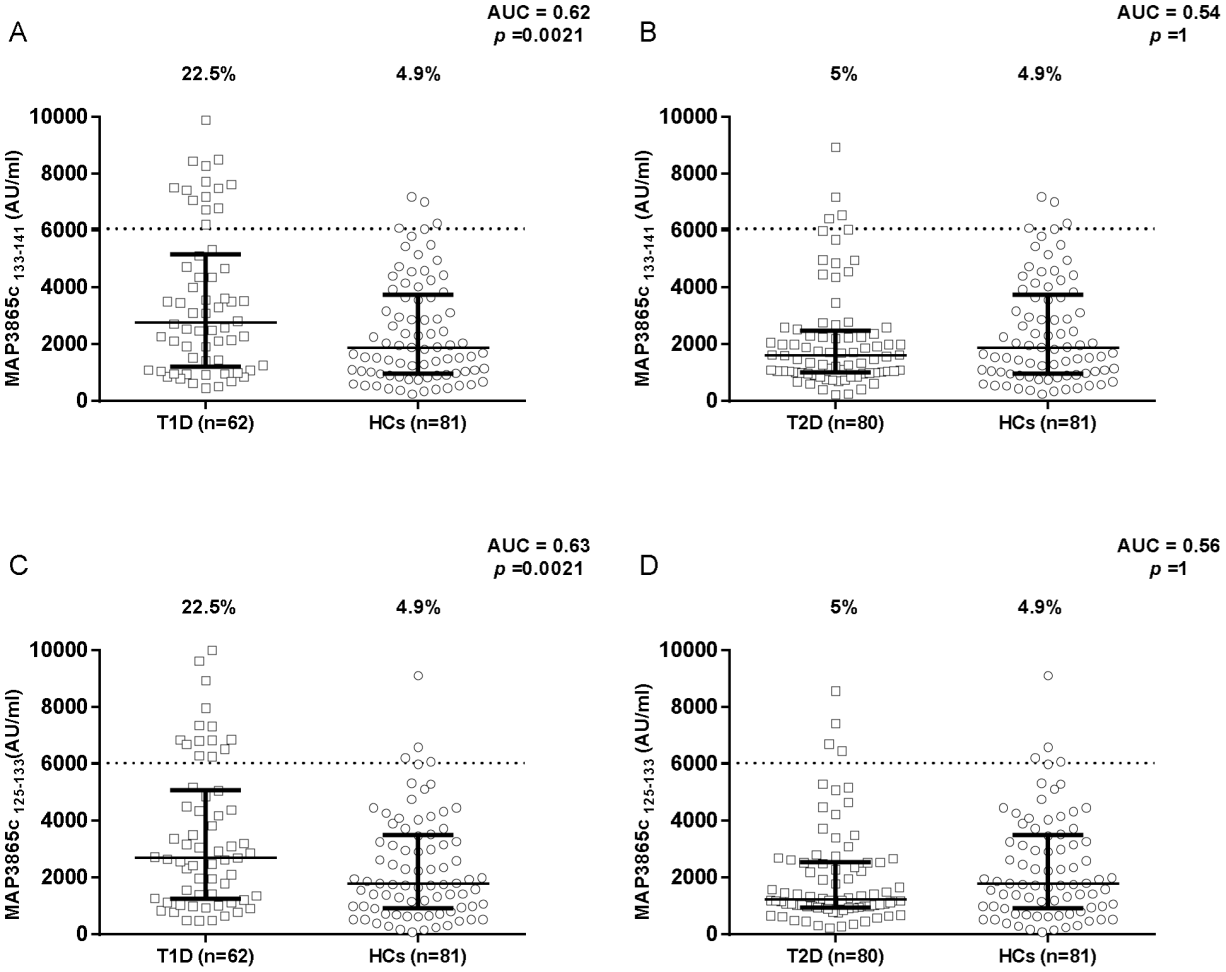
Prevalence of anti-MAP3865c antibodies in T1D and T2D patients with high-risk PDR and in healthy controls. Sera were tested for their reactivity against plate-coated MAP3865c_133–141_ (A and B) and MAP3865c_125–133_ (C and D) peptides. Antibody distribution is shown for T1D (A and C) and T2D (B and D) patients compared to controls. In each essay, the dotted line indicates the cut-off for positivity, as calculated by ROC analysis. The percent fraction of antibodies + sera is indicated on the top of each distribution, while bars indicate the corresponding median-interquartile range. AUC and *p* values determined by Fisher exact test are shown in the top right corner.

**Figure 2:**
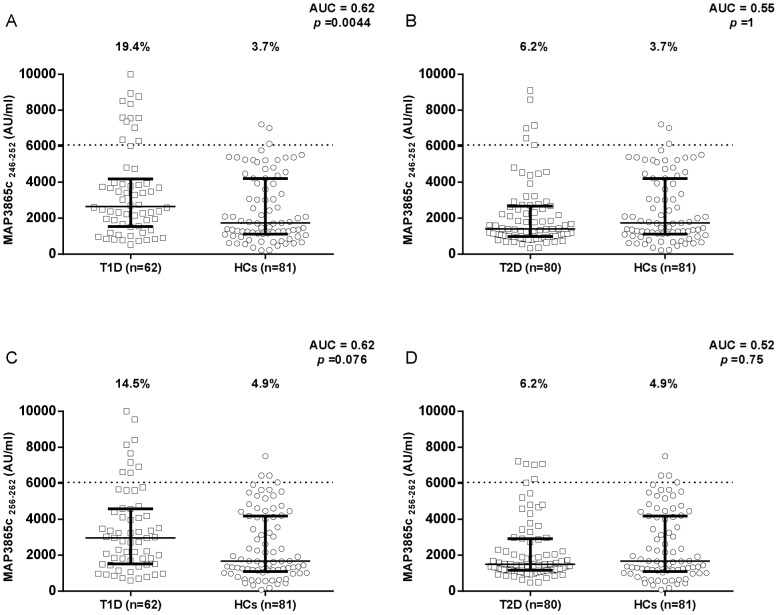
Prevalence of anti-MAP3865c antibodies in T1D and T2D patients with high-risk PDR and in healthy controls. Sera were tested for their reactivity against plate-coated MAP3865c_246–252_ (A and B) and MAP3865c_256–262_ (C and D) peptides. Antibody distribution is shown for T1D (A and C) and T2D (B and D) patients compared to controls. Data representation is the same as in Fig. 1.

**Figure 3:**
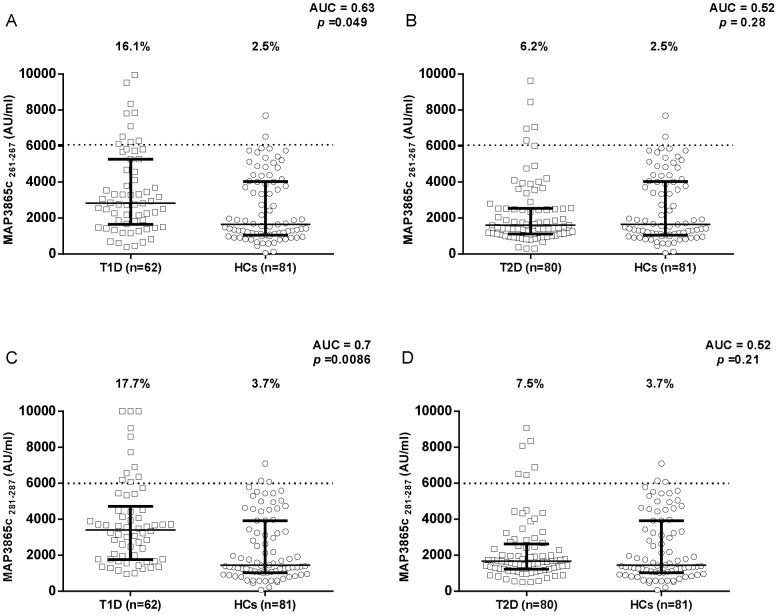
Prevalence of anti-MAP3865c antibodies in T1D and T2D patients with high-risk PDR and in healthy controls. Sera were tested for their reactivity against plate-coated MAP3865c_261–267_ (A and B) and MAP3865c_281–287_ (C and D) peptides. Antibody distribution is shown for T1D (A and C) and T2D (B and D) patients compared to controls. Data representation is the same as in Fig. 1.

Results on the detection of antibodies against MAP/ZnT8 peptides in T1D and T2D patients compared to healthy controls are summarized in Figures 1, 2, 3. All six peptides were highly recognized and showed detectable reactivity. T1D patients had significantly higher rates of positive antibodies than the control subjects for 5 out of the 6 MAP/ZnT8 peptides tested. On the other hand, no statistically significant differences were found between T2D patients and healthy controls.

**Figure 4:**
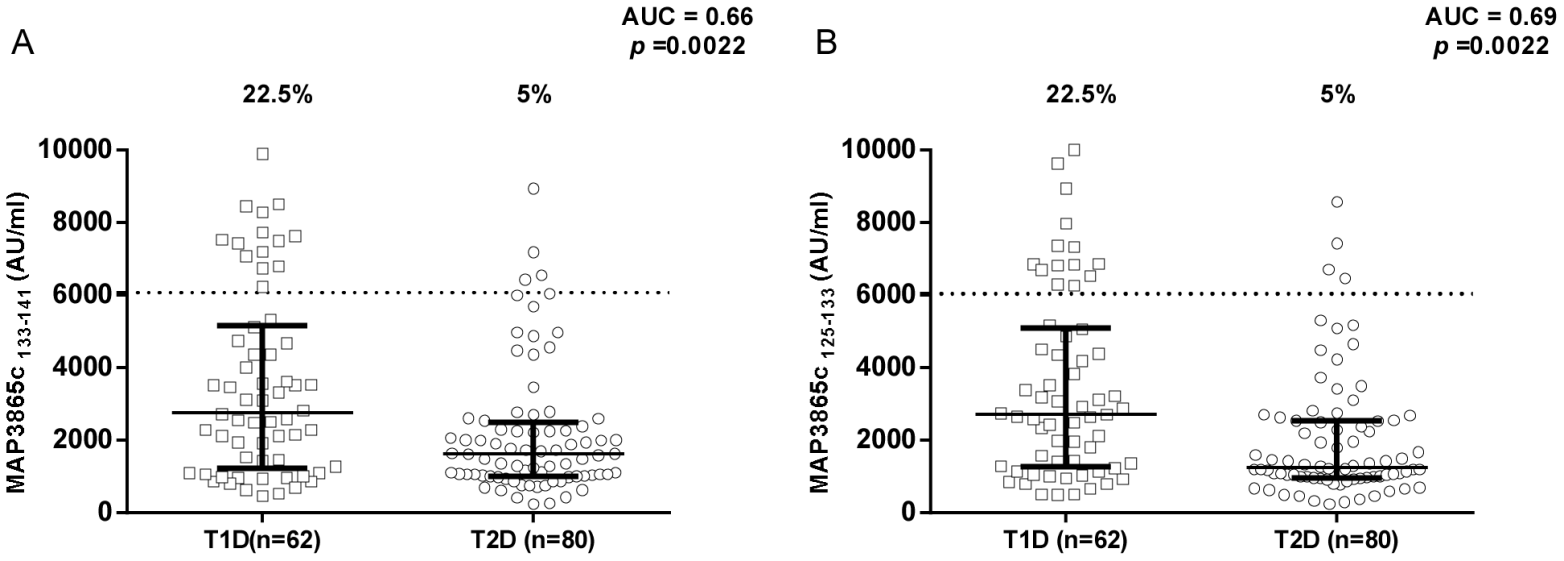
Prevalence of anti-MAP3865c antibodies in T1D and T2D patients with high-risk PDR. Sera were tested for their reactivity against plate-coated MAP3865c_133–141_ (A) and MAP3865c_125–133_ (B) peptides. Antibody distribution is compared between T1D and T2D patients. Data representation is the same as in Fig. 1.

**Figure 5:**
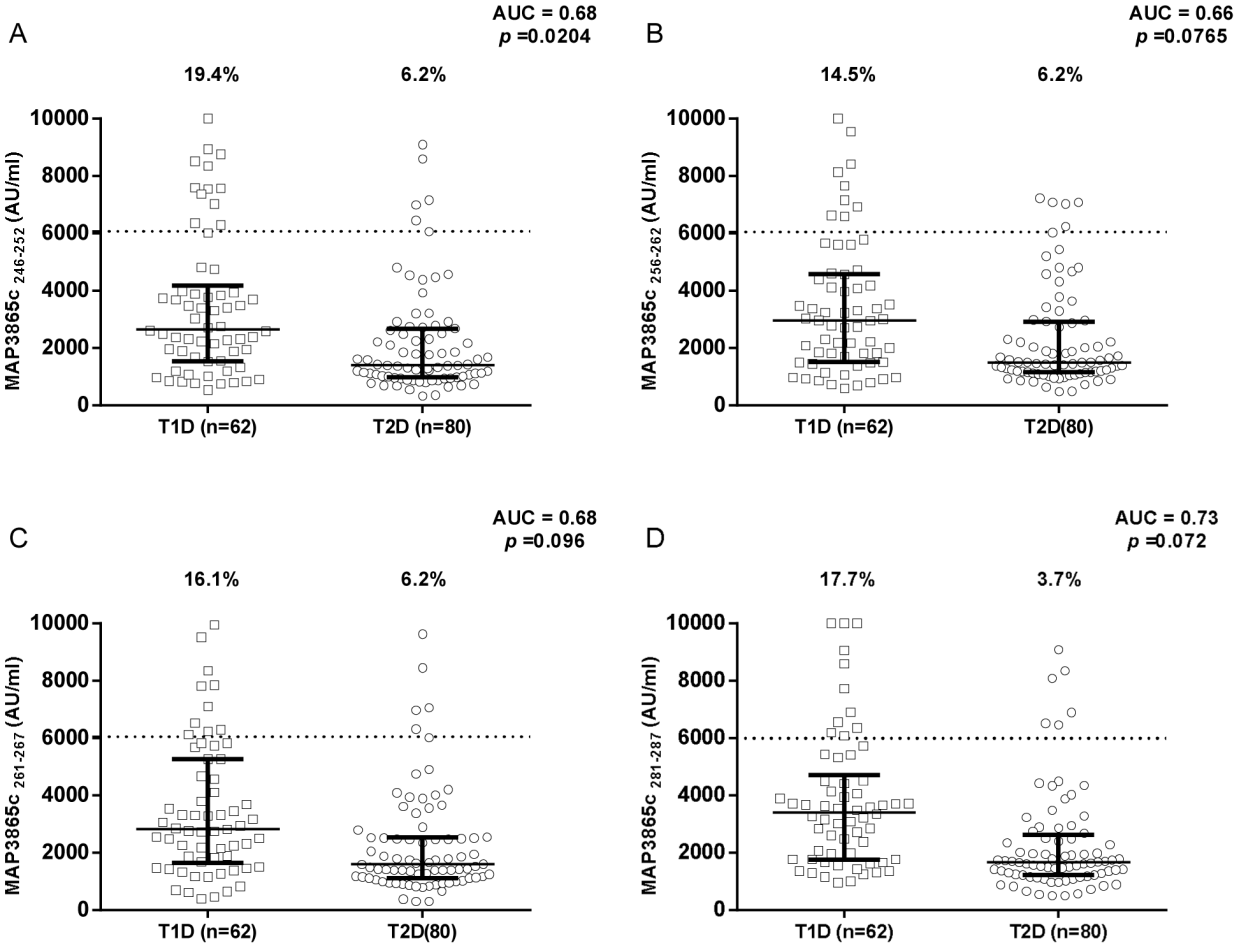
Prevalence of anti-MAP3865c antibodies in T1D and T2D patients with high-risk PDR. Sera were tested for their reactivity against plate-coated MAP3865c_246–252_ (A), MAP3865c_256–262_ (B), MAP3865c_261–267_ (C), and MAP3865c_281–287_ (D) peptides. Antibody distribution is compared between T1D and T2D patients. Data representation is the same as in Fig. 1.

In Figures 4 and 5, results on antibodies against MAP/ZnT8 peptides in T1D and T2D patients are compared. T1D patients showed significantly higher rates of positive antibodies against MAP3865c_125–133_, MAP3865c_133–141_, and MAP3865c_246–252_.

After categorization of T1D patients into two groups, one with positive, the other with negative antibodies against MAP/ZnT8 peptides, we found that both had similar systemic characteristics, with the exception of Hashimoto's thyroiditis, whose prevalence was 4/13 (30.7%) in the positive antibody group and 1/49 (2%) in the negative antibody group, a statistically significant difference (*P* = 0.016). No antibodies against MAP/ZnT8 peptides were found in the only T2D patient with Hashimoto's disease.

Mean visual acuity was 0.65±0.36 (range: 0–1) in T1D patients and 0.57±0.36 (range: 0–1) in T2D patients, a not statistically significant difference. Furthermore, T1D and T2D patients also had almost identical mean intraocular pressure values (14.9±2.4 mm Hg and 15.3±4.1 mm Hg, respectively). Twelve (19.4%) T1D patients and 20 T2D patients (25%) had a posterior chamber intraocular lens. Vitreous hemorrhage was observed in 18 (29%) T1D and in 20 (25%) T2D patients. After categorization of T1D patients into two groups, one with positive, the other with negative antibodies against MAP/ZnT8 peptides, we found that they had similar mean BCVA (0.67±0.36 and 0.61±0.35, respectively) and identical rates of vitreous hemorrhage (28.6%).

## Discussion

T1D is one of the most common autoimmune disorders, characterized by a T cell-mediated destruction of insulin-producing beta cells in the pancreas. This condition is believed to result from the interaction between multiple genetic and environmental factors, many of which are still poorly understood [13]. Former studies have shown that auto-antibodies against ZnT8 are common in newly-diagnosed cases of T1D [5], thus suggesting that this beta-cell membrane protein may act as a major autoantigen in T1D [4]. More recent investigations have found that antibodies against MAP3865c epitopes cross-react with ZnT8 epitopes [4], [9], [10], a result which could imply molecular mimicry between mycobacterial and pancreas beta-cell epitopes and raises the interesting question of whether MAP infection may be a potential environmental trigger for T1D. This finding, originally seen in T1D patients from Sardinia [4], [9], [10], an Italian island in the middle of the Mediterranean Sea, was later also observed in T1D patients from mainland Italy [8], [10].

There is a lot of experimental evidence indicating that Zn++ is abundant in the retina, where it plays an essential role in cell survival and in the normal functioning of antioxidant enzymes [6], [14]–[16]. Recently, dysregulation in Zn++ homeostasis caused by ZnT8 downregulation has been implicated in the pathogenesis of ischemic retinopathy [6]. Therefore, it is theoretically possible that ZnT8 downregulation might also be involved in the pathogenesis of PDR, because retinal ischemia caused by capillary occlusion is an essential step in the development of this vision-threatening vascular disorder. The detection of antibodies against MAP3865c epitopes cross-reacting with ZnT8 epitopes, associated with a more severe form of PDR with lower visual acuity and higher prevalence of vitreous hemorrhage, might provide an indirect sign of a possible correlation between ZnT8 dysregulation and DR. In this survey, in order to investigate this hypothesis, serum antibodies against 6 MAP/ZnT8 peptides were detected in high-risk PDR patients and healthy controls. We found that T1D patients had significantly higher rates of positive antibodies than the control subjects, a result consistent with former studies [4], [9], [10]. Similarly, T1D patients showed significantly higher rates of positive antibodies than T2D patients. On the other hand, no statistically significant differences were found between T2D patients and controls. Overall, these results confirm the association between T1D and the detection of antibodies against MAP/ZnT8 peptides, thus corroborating the hypothesis that MAP infection may be a potential environmental trigger for T1D.

In this study, T1D and T2D patients with high-risk PDR showed similar mean values of BCVA and had similar rates of vitreous hemorrhage. Likewise, after categorization of T1D patients into two groups, one with positive, the other with negative antibodies against MAP/ZnT8 peptides, we found that they had similar mean BCVA and identical rates of vitreous hemorrhage. On the whole, the absence of a more severe form of high-risk PDR in patients with positive antibodies against MAP/ZnT8 peptides might imply that auto-immune ZnT8 dysregulation plays a minor or no role in the pathogenesis of high-risk PDR.

One could argue that our choice of using BCVA and vitreous hemorrhage as ways of categorizing the severity degree of high-risk PDR might be questionable. Indeed, BCVA may be affected by concomitant macular edema and/or ischemia and cataract, all of which are common in patients with diabetic retinopathy. However, there is little doubt that the more serious forms of high-risk PDR are associated with reduced BCVA and vitreous hemorrhage. Therefore, we feel strongly that these parameters can provide a measure of the severity of high-risk PDR.

An unexpected result of post-hoc analysis was the significantly higher prevalence of Hashimoto's thyroiditis in the group of T1D patients with positive antibodies against MAP/ZnT8 peptides. To the best of our knowledge, we are unaware of any published study reporting a similar association. This finding is intriguing and raises the interesting questions of whether T1D and Hashimoto's disease share a common pathogenic mechanism and whether T1D patients with positive antibodies against MAP/ZnT8 peptides might have a higher risk of developing auto-immune thyroiditis.

In a recent population-based study exploring the prevalence and comorbidity of 12 auto-immune diseases in Sardinia, Sardu et al. disclosed that Hashimoto's thyroiditis is the most common autoimmune disease and that individuals affected by one autoimmune disorder are more likely to develop a second one [17]. Furthermore, experimental and clinical evidence indicates that MAP may be a potential environmental trigger of Hashomoto's thyroiditis, as suggested by the detection of viable MAP organism and serum antibodies against MAP/ZnT8 peptides in patients suffering from this auto-immune thyroid disease [18]–[20].

In ruminants, MAP specifically colonizes the mucosa-associated lymphoid tissue (MALT) of the small intestine, where this organism grows and multiplies within the intraepithelial macrophages, thus causing Johne's disease (JD) [21]. In humans, MAP is transmitted by the ingestion of contaminated dairy products and causes an asymptomatic infection, which has been associated not only with T1D and Hashimotos's thyroiditis, but also with Crohn's disease [22] and multiple sclerosis [23]. Overall, all these data support the idea that a common pathogenic mechanism may be responsible for these different autoimmune diseases.

Recent research has shown increased serum concentrations of CXCL10 chemokine in T1D children with antibodies against MAP3738c protein [24]. Furthermore, high circulating CXCL10 levels associated with T-helper (Th) 1 autoimmune response have been observed in new onset T1D and Hasmimoto's disease [25], [26]. Therefore, the significantly higher prevalence of Hashimoto's thyroiditis among T1D patients with positive antibodies against MAP/ZnT8 might in part be due to a common immuno-pathogenic mechanism involving Th 1 immune response and chemokines.

Our study has several important limitations. First, it was restricted to a limited, genetically homogeneous group of patients (i.e. those of Sardinian ancestry); as a result, our findings may not be applicable to diabetic patients of non-Sardinian ancestry. Second, we analyzed a relatively small number of subjects. Third, we do not know whether T1D patients with positive antibodies against MAP/ZnT8 peptides developed high-risk PDR earlier than those with negative antibodies. Fourth, in patients suffering from Hashimoto's disease, we originally did not collect sufficiently detailed information about the thyroid condition, as the primary goal was to investigate the role of antibodies against MAP/ZnT8 peptides in the pathogenesis of high-risk PDR. Last, but not least, we compared T1D and T2D patients with high-risk PDR to normal subjects, rather than to diabetic controls without diabetic retinopathy. This second, potentially more informative comparison will be the subject of further investigation.

In conclusion, this study confirmed that T1D patients have significantly higher rates of positive antibodies against MAP/ZnT8 peptides, but failed to find a correlation between their presence in the patients' serum and the degree of severity of high-risk PDR. On the other hand, we found a significantly higher prevalence of Hashimoto's thyroiditis in the group of T1D patients with positive antibodies. At this stage, no definitive conclusion can be drawn from our preliminary results. Further and larger clinical and experimental trials are required to clarify the exact role of antibodies against MAP/ZnT8 peptides in the pathogenesis of PDR in T1D and to establish whether, or not, T1D patients are more susceptible to Hashimoto's disease.
